# Sex-dependent factors of alcohol and neuroimmune mechanisms

**DOI:** 10.1016/j.ynstr.2023.100562

**Published:** 2023-08-03

**Authors:** Bryan Cruz, Vittoria Borgonetti, Michal Bajo, Marisa Roberto

**Affiliations:** a Department of Molecular Medicine, The Scripps Research Institute, La Jolla, CA, USA, 92073

**Keywords:** Alcohol, Neuroimmune, Cytokine, Microglia, Astrocyte, Sex differences

## Abstract

Excessive alcohol use disrupts neuroimmune signaling across various cell types, including neurons, microglia, and astrocytes. The present review focuses on recent, albeit limited, evidence of sex differences in biological factors that mediate neuroimmune responses to alcohol and underlying neuroimmune systems that may influence alcohol drinking behaviors. Females are more vulnerable than males to the neurotoxic and negative consequences of chronic alcohol drinking, reflected by elevations of pro-inflammatory cytokines and inflammatory mediators. Differences in cytokine, microglial, astrocytic, genomic, and transcriptomic evidence suggest females are more reactive than males to neuroinflammatory changes after chronic alcohol exposure. The growing body of evidence supports that innate immune factors modulate synaptic transmission, providing a mechanistic framework to examine sex differences across neurocircuitry. Targeting neuroimmune signaling may be a viable strategy for treating AUD, but more research is needed to understand sex-specific differences in alcohol drinking and neuroimmune mechanisms.

## Abbreviations

5-HTT: 5-hydroxytryptamine (serotonin) transporter
ASC: adaptor protein apoptosis speck-like protein
AUD: Alcohol use disorder
B2m: β-2-microglobulin
BNST: bed nucleus of the stria terminalis
Cd14: CD14 molecule
CFA: complete Freundʼs adjuvant
CHRM2: muscarinic acetylcholine receptor M2
CHRM5: muscarinic acetylcholine receptor M5
CHRNA5: neuronal acetylcholine receptor subunit α5
CHRNB2: neuronal acetylcholine receptor subunit β2
CIE: chronic intermittent ethanol
CNS: central nervous system
COX-2: cyclooxygenase-2
CSF: colony-stimulating factor
Ctsf: cathepsin F
Ctss: cathepsin S
DID: drinking-in-dark
DRD2: dopamine D_2_ receptor
GABRA1: γ-aminobutyric acid receptor subunit α1
GABRB2: γ-aminobutyric acid receptor subunit β2
GFAP: glial fibrillary acidic protein
GRM8: metabotropic glutamate receptor 8
HPC: hippocampus
HTR1B: 5-HT_1B_ receptor
HTR3A: 5-HT_3A_ receptor
HTR3B: 5-HT_3B_ receptor
IBA-1: ionized calcium-binding adapter molecule-1
IFN: interferon
IL-1ra: interleukin-1 receptor antagonist
IL: interleukin
Il1rn: interleukin 1 receptor antagonist
Il6: interleukin 6
Il18: interleukin 18
iNOS: inducible nitric oxide synthase
LPS: lipopolysaccharide
MAPK: mitogen-activated protein kinase
MCP1/CCL2: monocyte chemoattractant protein 1/chemokine ligand 2
MPDZ: multiple PDZ domain protein
NAc: nucleus accumbens
NCAM1: neural cell adhesion molecule 1
NRPL1: nucleotide-binding, leucine-rich repeat pyrin domain containing protein 1
NRPL3: nucleotide-binding, leucine-rich repeat pyrin domain containing protein 3
NF-κB: glial nuclear factor κ-light-chain-enhancer of activated B cells
P2X7: purinoreceptor 7
PCSK9: proprotein convertase subtilisin/kexin 9
PDE4: phosphodiesterase-4
PEA: palmitoylethanolamide
PFC: prefrontal cortex
Ptn: pleiotrophin
RPTP β/ζ: receptor protein tyrosine phosphate
SLC6A4: sodium-dependent serotonin transporter
TGF: transforming growth factor
TLR-4: Toll-like receptor 4
TNF: tumor necrosis factor
TSPO: translocator protein
TTC12: tetratricopeptide repeat domain 12
VS: ventral striatum

## 1 Introduction

Alcohol use disorder (AUD) is a major health problem worldwide. Compelling evidence indicates that sex/gender plays a crucial role in the development and trajectory of AUD. Although men are generally known to consume higher amounts of alcohol, alcohol consumption in women over the past decade has dramatically increased in the United States, with patterns of drinking that are now close to men (Grant et al., 2017; Guinle and Sinha, 2020). Importantly, factors that promote AUD between men and women are distinct. For example, women are more likely to self-medicate with alcohol to avoid negative affect, and they experience more emotional distress, including social trauma, anxiety, and depression, than men (Guinle and Sinha, 2020; Erol and Karpyak, 2015; Sinha, 2008; Fox and Sinha, 2009). This heightened stress sensitivity in women is a strong predictor of heavy alcohol drinking and other alcohol-related health problems (Erol and Karpyak, 2015). Thus, it is critical to understand the neurobiology that promotes sex differences in AUD.

The role of neuroimmune signaling in AUD has received significant attention. AUD impairs neuroimmune signaling. Genetic polymorphism in neuroimmune genes including cytokines, microglia, and astrocytes is associated with the innate predisposition to heavy alcohol drinking in humans and rodents (Pastor et al., 2005; Marcos et al., 2008; Sery et al., 2003; Crews and Vetreno, 2014; Rupprecht et al., 2010; Ponomarev et al., 2012). Clusters of enriched neuroimmune-related genes (e.g., *Il1r1*, *Il1rn*, *Cd14*, and *Il10rb*) have been associated with alcohol preference across different mouse models and genotypes (Ponomarev et al., 2012; Blednov et al., 2012, 2015; Marshall et al., 2016; Tabarean et al., 2006; Iancu et al., 2013; Farris et al., 2015). Animal studies that used transgenic and pharmacological approaches to target neuroinflammatory pathways have found alterations in alcohol drinking across several drinking paradigms (Blednov et al., 2012, 2017a, 2017b; Lovelock et al., 2022; Gruol et al., 2023; Bajo et al., 2016; Harris et al., 2017). Recent findings in humans and rodents suggests a role for sex differences in both AUD and neuroimmune signaling (Hitzemann et al., 2022; West et al., 2021; Warden et al., 2019a). However, few studies have reported quantitative sex differences across multiple levels of investigation.

The present review discusses studies that report 1) genomic and 2) transcriptomic evidence on neuroimmune systems following alcohol exposure in animal models and associations of gene polymorphisms in humans, 3) molecular and 4) synaptic evidence, and 5) behavioral evidence of alcohol intake by pharmacological manipulations of specific neuroimmune systems in the context of sex differences. This review focuses on cytokines and immune cells (microglia, astrocytes) as mediators of alcohol-induced neuroimmune responses and modulators of neurotransmission. Lastly, we highlight evidence of sex differences in neuroimmune systems following alcohol exposure, which may have therapeutic importance for the treatment of AUD.

### 1.1 Definitions & literature search

This review conducted a comprehensive literature search via PubMed and Google Scholar databases. Importantly, this review primarily highlights male and female studies in rodents and humans. However, we also indicate studies conducted only in males that have strong potential or provide a foundational bases to be studied in females at each level of investigation. In the instance that we did not identify studies conducted in both male and female subjects, we specifically note the lack of sex difference at each level of investigation. We also highlight that this report is not an exhaustive review but focuses on summarizing critical literature that sheds light on sex-dependent factors of neuroimmune mechanisms and alcohol-related behaviors. The following terms were used for all searches: alcohol, alcohol dependence, alcohol drinking, sex differences, male (s), female (s), mice, rat (s), cytokine, microglia, astrocyte, synaptic transmission, genomic, transcriptomic, neuroinflammation, neuroimmune response, neuroimmune signaling, and neuroinflammation system (s). We define neuroinflammation as an innate immune inflammatory response after toxic insult from alcohol use via production of cytokines in the CNS. Neuroimmune signaling pertains to signaling molecules that behave as neuromodulators stemming from the innate immune system to alter synaptic function and/or neuroplasticity, which plays a crucial role in regulating various physiological and pathological processes in CNS cell types (neurons, microglia, and astrocytes). The term neuroimmune systems refers to a general communication of the CNS and the immune system to mechanistically influence cellular function or behavioral output. Lastly, this review does not focus on how alcohol exerts its biological effects on neuroimmune systems. For detailed reviews on basic biological functions on alcohol and neuroimmune systems the readers are directed to specific reviews (Crews and Vetreno, 2014; Roberto et al., 2018).

### 1.2 Genomic evidence

Genomic findings on alcohol and neuroimmune signaling have provided important insights into complex interactions between alcohol consumption and the immune system. Human genomic studies have revealed polymorphisms of genes that are associated with alcohol dependence and act on alcohol-relevant neurotransmitter signaling, such as γ-aminobutyric acid (GABA) receptor subunit α1 (*GABRA1*), GABA receptor subunit α2 (*GABRA2*), GABA receptor subunit γ1 (*GABRG1*; i.e., GABAergic signaling), monoamine oxidase (*MAOA*), catechol-*O*-methyltransferase (*COMT*), and dopamine D_2_ receptor (*DRD2*), *ANKK1*, tetratricopeptide repeat domain 12 (*TTC12*), and neural cell adhesion molecule 1 (*NCAM1*; dopaminergic signaling), 5-hydroxytryptamine (serotonin) transporter (*5-HTT*), sodium-dependent serotonin transporter (*SLC6A4*), 5-HT_3A_ receptor (*HTR3A*), 5-HT_3B_ receptor (*HTR3B*), 5-HT_1B_ receptor (*HTR1B*; i.e., serotonergic signaling), metabotropic glutamate receptor 8 (*GRM8*; i.e., glutamatergic signaling), muscarinic acetylcholine receptor M2 (*CHRM2*), neuronal acetylcholine receptor subunit α5 (*CHRNA5*), and neuronal acetylcholine receptor subunit β2 (*CHRNB2*; i.e., cholinergic signaling) (Luo et al., 2005; Ehringer et al., 2007; Wang et al., 2009; Kohnke et al., 2005; Yang et al., 2008; Tikkanen et al., 2009; Hendershot et al., 2012; van der Zwaluw et al., 2010; Cao et al., 2013; Seneviratne et al., 2013; Agrawal et al., 2006; Dick and Bierut, 2006; Enoch, 2008; Chen et al., 2009; Tabakoff et al., 2009). Genome-wide association studies have identified several genes that are associated with AUD and involved in immune system function, including genes that are related to cytokine signaling, T-cell activation, and antigen presentation (Gelernter et al., 2014; Kapoor et al., 2013). Additionally, epigenetic modifications have been found to play a role in the neuroimmune response to alcohol exposure. For example, chronic alcohol consumption alters brain DNA methylation and histone modifications, leading to alterations of the expression of genes that are involved in immune function and inflammation (Pandey et al., 2008). Despite growing evidence, most studies have generally been limited to males and have not addressed the role of sex in genetics of AUD. A previous genomic study in humans revealed genetic associations with single-nucleotide polymorphisms (SNPs) of the glutamate decarboxylase 1 (*GAD1*), multiple PDZ domain protein (*MPDZ*), muscarinic acetylcholine receptor M5 (*CHRM5*), GABA receptor subunit β2 (*GABRB2*), *MAPK1*, and protein phosphatase 1 regulatory subunit 1B (*PPP1R1B*) genes following alcohol consumption (Tabakoff et al., 2009). This report includes a subset of male and female subjects in their analysis (Montreal cohort), but only *MPDZ* was significantly altered when including both males and females in the findings (Tabakoff et al., 2009). Changes in other genomic targets for the latter report were primarily evaluated in male subjects. Studies in this area on sex differences are limited and, to our knowledge, the interaction between estrous cyclicity/hormone status on the expression of these genomic drivers following alcohol exposure warrants further investigation.

### 1.3 Transcriptomic evidence

Transcriptomic studies have also provided compelling evidence that sex differences contain unique neuroimmune signatures following alcohol consumption (for a detailed review, see (Hitzemann et al., 2022)). Despite receiving a similar concentration of acute alcohol administration, males and females exhibit distinct changes in their transcriptomes. This divergent effect suggests approximately 10% overlap between male and female genes, suggesting distinct transcriptional differences among sexes (Hitzemann et al., 2022; Agrawal et al., 2014). In a binge alcohol drinking study, changes in gene expression were observed in reward-related regions (i.e., NAc) in male and female B6 mice after repeated 24-h alcohol access sessions (Finn et al., 2018). Of the 384 genes that were collected, the authors found sex differences for 30 genes following binge-like drinking behavior (Finn et al., 2018). This analysis found that binge-like drinking specifically altered hormone signaling and neuroimmune function in female mice and disrupted neurotransmitter metabolism pathways in male mice (Finn et al., 2018). Tumor necrosis factor receptor 2 (*TNFR2*) signaling also showed inactivation in females and activation in males, indicated by the expression of *Lta*, which encodes lymphotoxin-α (Finn et al., 2018). Moreover, reductions of *Il6* were observed in females and not in males following binge-like drinking behavior (Finn et al., 2018).

In rodents, microarray studies suggest that sex differences in gene profiles exist in AUD (Flatscher-Bader et al., 2008; Kimpel et al., 2007; Mulligan et al., 2006; Liu et al., 2006). Based on these previously published genomic datasets, a follow-up study selectively tested candidate genes in alcohol-related behaviors (Blednov et al., 2012). Using mutant male and female mice, this study examined six potential candidate genes, including β-2-microglobulin (*B2m*), cathepsin S (*Ctss*), cathepsin F (*Ctsf*), interleukin 1 receptor antagonist (*Il1rn*), CD14 molecule (*Cd14*), and interleukin 6 (*Il6*), following alcohol drinking using several drinking paradigm (Blednov et al., 2012). This study found that the lack of *B2m*, *Ctss*, *Ctsf*, *Il1rn*, *Il6*, and *Cd14* reduced alcohol preference and intake in a paradigm- and sex-specific manner (Blednov et al., 2012).

In females, the estrous cycle may influence the expression of neuroimmune changes and modulate alcohol drinking (DiCarlo et al., 2017). A study across multiple brain regions (hypothalamus, cerebellum, neocortex, and hippocampus) found 210 differentially expressed genes out of 16,000 genes, and 61 of those genes appeared to be responsive to estrogen in high alcohol-preferring mice (DiCarlo et al., 2017). Another study found that the medial PFC (mPFC) showed 935 differentially expressed genes from proestrus to diestrus, and these differentially expressed genes were associated with the modulation of synaptic transmission (Duclot and Kabbaj, 2015; Ford et al., 2002). A previous study also found that female and male dependent rats exhibited differences in blood-brain immune response modules in the transcriptomic network analysis. Alcohol-dependent females expressed *Cst5*, *mir-17*, *betulinic acid*, *Mmp 3*, *miR-17-5p*, *Nup107*, *Rbm5*, *Tlr4*, *Ly96*, *Sf3b1*, *Aim 2*, *Tnrc6a*, and *Abca 1*, and alcohol-dependent males expressed *Abcb6*, *1,2-dithiol 3-thione*, *Klf1*, *Rictor*, *St1926*, *Bnip3l*, *Fth 1*, *Cdc25b*, *Grp*, *Mrpl12*, *E2f1*, *Ola 1*, *Tpm 1*, *Fancd2*, *Ctsb*, *E2f2*, *Uba1*, *E2f3*, *Cul 1*, *Rb1cc1*, *Fzr1*, *Sub 1*, *Stx 2*, *Rb1*, *Gadd45a*, and *Gsk3a* versus their respective controls (Ferguson et al., 2022). Overall, these findings suggest sex differences in molecular mechanisms that underlie the neuroimmune response to alcohol use. Further research is needed to fully elucidate these mechanisms and develop sex-specific treatments for AUD.

### 1.4 Cytokine evidence

Cytokines are a group of more than 300 soluble glycoproteins, including tumor necrosis factors (TNFs), interleukins (ILs), chemokines, interferons (IFNs), colony-stimulating factors (CSFs), and transforming growth factor (TGF), that are produced by cells in response to immunological stimuli (e.g., microbes, toxins, and tissue damage). Cytokines play a crucial role in regulation of the innate and adaptive immune responses (Liu et al., 2021). Alcohol exposure significantly affects cytokines in animal models. For example, chronic alcohol exposure increases levels of pro-inflammatory cytokines (e.g., IL-1β and TNF-α) and other inflammatory mediators that are related to neurotoxicity (e.g., inducible nitric oxide synthase [iNOS], cyclooxygenase-2 [COX-2]) in both female and male rats (Alfonso-Loeches et al., 2013). In female mice, chronic alcohol exposure activates inflammasome components (NLRP1, NLRP3, ASC) along with increased caspasae-1 activity and IL-1β protein levels in the cerebellum and cortex (Lippai et al., 2013). This study also examined the role of inflammasome activation in alcohol-induced IL-1β, by using mice deficient in the inflammasome sensor, NLRP3, or the adaptor molecule, ASC (Lippai et al., 2013). Lippai et al. (2013) and observed no changes in IL-1β protein levels or caspase-1 activity in alcohol-exposed NLRP3 or ASC deficient female mice versus controls, suggesting that the lack of NLRP3 inflammasome or ASC are critical for alcohol-induced increase in IL-1β production in the brain (Lippai et al., 2013). Levels of iNOS and COX-2 are also higher in alcohol-exposed female rats than in male rats, suggesting greater susceptibility to neurotoxic effects of alcohol (Alfonso-Loeches et al., 2013). Another study found that chronic alcohol intake increased iNOS, COX-2, IL-1β, TNF-α, and IL-6 in the cerebral cortex in female wildtype mice (Alfonso-Loeches et al., 2010). This study also reported higher levels of CD11b (a microglial marker) and glial fibrillary acidic protein (GFAP; an astrocyte marker) in the same female wildtype mice, suggesting an important role for microglial and astrocytes following chronic alcohol exposure in females (Alfonso-Loeches et al., 2010).

Other compelling evidence indicating differences in cytokines between males and females after alcohol exposure. For example, studies employing lipopolysaccharide (LPS)-induced model of neuroinflammation, show that female LPS-treated rats consumed more alcohol than male rats (Decker Ramirez et al., 2023). Female but not male LPS-treated rats also exhibited significant increases in IL-6 in the prefrontal cortex (PFC) and ventral striatum (VS), two critical brain regions that mediate alcohol-associated behaviors (Decker Ramirez et al., 2023). TNFα levels also increased in the hippocampus (HPC) and decreased in the PFC of female versus male LPS-treated rats (Decker Ramirez et al., 2023). With regard to translational investigations, binge alcohol intoxication studies have also shown selective increases in the expression of several cytokines and chemokines in the PFC and plasma in female adolescent alcohol-exposed humans and mice (Pascual et al., 2017). Specifically, this study found that binge alcohol drinking increased peripheral IL-17A, monocyte chemoattractant protein 1/chemokine ligand 2 (MCP1/CCL2), and macrophage inflammatory protein 1α levels in both human and adolescent female but not male mice (Pascual et al., 2017). This same immune signaling also increased in the PFC in females but not males (Pascual et al., 2017). Other studies using a complete Freund's adjuvant (CFA) model of pain found alterations in cytokines and sex differences in several brain regions associated with alcohol-related behaviors. CFA-treated female rats but not male rats exhibited increases in IL-1β and decreases in IL-10 in the nucleus accumbens (NAc) during alcohol reinstatement behavior (Cuitavi et al., 2021).

Such actions and sex differences in cytokines are also observed in peripheral systems. In humans, meta-analysis review has revealed that higher concentrations of plasma TNFα, IL-1β, IL-6, IL-8, IL-10 versus healthy control subjects, however, very few studies compare sex differences likely due to a limited number of female samples obtained in studies (Moura et al., 2022; de Timary et al., 2017). Rat studies that used a alcohol liquid diet or sucrose found higher hepatic levels of TNFα and IL-6 in alcohol-exposed rats (Colantoni et al., 2003). Interestingly, hepatic levels of TNFα were higher in female versus male alcohol-exposed rats, whereas hepatic IL-6 levels only increased in alcohol-exposed males (Colantoni et al., 2003). Intragastric alcohol administration also increased hepatic mRNA and protein expression of the IL-6a receptor and increased the phosphorylation of signal transducer and activator of transcription 3 (STAT3; a downstream signaling pathway of several cytokine receptors) in females versus males at 2 and 4 weeks of exposure (Gallucci et al., 2004). Overall, these findings demonstrate sex differences in cytokine production following alcohol exposure in both the CNS and periphery, which may contribute to females being more vulnerable to neurotoxic/neuroinflammatory effects of alcohol. We note that studies involving estrous cycle to influence alcohol-related cytokine alterations are limited. We acknowledge only one report showing that the combination of alcohol and estradiol supplementation increased hepatic protein levels of IL-6 and TNFα in female rats (Lee et al., 2012).

### 1.5 Microglial evidence

Recent research has identified several important roles of microglial cells in the brain following alcohol exposure and associated behavioral responses (Roberto et al., 2018; Stellwagen et al., 2019). Microglia play a key role in maintaining CNS homeostasis, are the main immune cells in the CNS, and function to respond to tissue injury and infection (Ben Achour and Pascual, 2010; Li et al., 2021; Subhramanyam et al., 2019). Emerging evidence suggests that microglia have promising therapeutic efficacy for various neurological diseases, based on their ability to modulate, repair, and remodel local neuronal cell types (Subhramanyam et al., 2019; Szepesi et al., 2018). Chronic pathological conditions, such as AUD, can alter microglial “reactive” state, leading to toxic neuroinflammation (Paolicelli et al., 2022; Takeuchi, 2010). Much evidence indicates that microglia are key factors in the trajectory of AUD (He and Crews, 2008; Marshall et al., 2013; Walter and Crews, 2017; Warden et al., 2020). Indeed, microglia respond to neurotoxic effects of chronic alcohol exposure as previously observed in several *in vitro* and *in vivo* animal models (Agrawal et al., 2011, 2014; Lawrimore et al., 2019). Four-day alcohol binge cycles increases expression of M1 and M2 microglia markers in rats (Peng et al., 2017). M1 microglia are known to produce neurotoxic effects via production and release of pro-inflammatory cytokines, chemokines, and reactive oxygen species, and M2 microglia have neuroprotective function and is associated with production of anti-inflammatory cytokines (Peng et al., 2017; Benarroch, 2013). Moreover, levels of translocator protein (TSPO), a biomarker of microglial activation, increased in the amygdala in postmortem brains of AUD patients (Rupprecht et al., 2010; Ponomarev et al., 2012). Chronic alcohol exposure promotes microglial activation via several receptor families (e.g., Toll-like receptor 4 [TLR-4] and purinoreceptor 7 [P2X7]) and transcription factors (e.g., glial nuclear factor κ-light-chain-enhancer of activated B cells [NF-κB]). This, in turn, leads to the release of pro-inflammatory mediators (e.g., IL-1β and IL-6), an immune response that is elicited by chronic alcohol and known mediators of AUD (Harris et al., 2017; Bajo et al., 2014; Asatryan et al., 2018; Patel et al., 2019, 2022a; Roberts et al., 2019). Lastly, bioinformatics analyses of brain tissue from animals that underwent binge alcohol drinking revealed differences in neuroimmune responses including the upregulation of TLR, mitogen-activated protein kinase (MAPK), Jak-STAT, and chemokine signaling, which are highly related to microglia (Agrawal et al., 2014).

Importantly, the interaction between sex differences in microglia and alcohol drinking is limited. However, strong evidence suggests that sex differences may impact the microglial state in different pathological disorders, providing further support for studying microglia as potential treatment targets for AUD. Transcriptomic profiling of microglia has revealed sex-dependent differences in adult mice (Villa et al., 2018). Proteomic analysis of microglia isolated from male and female brains after 4 months of alcohol exposure found sex specific difference in various proteins (Rath et al., 2020). Most studies reporting that microglia inhibition decreased alcohol consumption were mainly performed in males. For example, in a voluntary choice drinking model, the administration of minocycline (50 mg/kg), a second-generation tetracycline antibiotic (microglia suppressor), decreased alcohol intake in both males and females (Agrawal et al., 2011). Similarly, in the study using the drinking-in-dark (DID) paradigm, minocycline reduced DID in adult male and female mice but not in adolescent mice of either sex, providing evidence of the age-divergent role of neuroimmune pathways in regulating alcohol consumption (Agrawal et al., 2014). Importantly, minocycline can suppress brain microglial activation evident by reduced co-localization of ionized calcium-binding adapter molecule-1 (IBA-1) and class D (CD) 68 in positive microglia cells in both naïve and alcohol-exposed mice (Karelina et al., 2018; Bassett et al., 2021). These actions of minocycline may occur through other mechanisms that interact with microglia, since one study found that alcohol and stress exposure reduced brain 5-HT expression and minocycline prevented loss of 5-HT expression in microglial IBA-1 expressing cell types, suggesting inhibiting microglia to dampen alcohol drinking may occur through serotonergic systems (Lee et al., 2021). Compounds that are structurally similar to minocycline, such as tigecycline, reduced alcohol intake in both dependent and non-dependent female and male mice at high doses (80 and 100 mg/kg) (Bergeson et al., 2016; Martinez et al., 2016; Syapin et al., 2016). The mechanism by which tigecycline reduces alcohol intake are unclear with no direct link to suppress microglial activation (Bergeson et al., 2016). Barton et al. reported that females had significantly more microglial activation in the HPC, whereas males had fewer microglia following alcohol drinking (Barton et al., 2017). In addition to the brain, chronic alcohol exposure induces microglial activation also in the spinal cord. Recently, Borgonetti et al. (2023) found an increase in IBA-1, and CSF-1, IL-6, and ERK44/42 in male and female mice with pain-related hypersensitivity from alcohol abstinence in spinal cord tissue (Borgonetti et al., 2023a). These immune markers assessed in Borgonetti et al. (2023) are critical for microglial viability (CSF-1), and promote calcium binding in microglia cells (IBA-1), and IL-6 has been shown to be potent inducer and modulator of microglial activation (Warden et al., 2020; Elmore et al., 2014; Lituma et al., 2021; Zhou et al., 2016). Both alcohol-dependent males and females also developed strong mechanical allodynia during alcohol withdrawal, which was then normalized after voluntary drinking, suggesting alleviation by alcohol exposure in both sexes (Borgonetti et al., 2023a). However, the magnitude of the change in hypersensitivity to the mechanical stimulus after acute alcohol drinking was greater in females versus males. This difference may be related to higher alcohol consumption observed in the females, which suggest female vulnerability to alcohol is associated with motivation to alleviate alcohol-evoked neuropathic pain (Borgonetti et al., 2023a).

Overall, increases in the number and activation of microglia in females demonstrate a sex difference in the microglia response to alcohol, which may have implications for pharmacotherapies to treat AUD. We consider that estrous cyclicity and hormonal status may influence microglial changes after heavy alcohol, an area understudied and requires future work. One transcriptome study revealed that microglia in females produce a more protective characteristic and restricts damage following insult by cerebral ischemia than male mice (Villa et al., 2018). The authors in this report suggested that the neuroprotective effects and difference in microglia found in females appear to be a result of inherent production of sex steroids/hormones/organs assigned at birth (Villa et al., 2018). This may be due to local neonatal synthesis of estrogen associated with the androgen surge from the male testis that might produce organizational effects on these cells by inducing a sex-specific phenotype in adult mice (Villa et al., 2018). Importantly, the emerging neuroinflammatory target proprotein convertase subtilisin/kexin 9 (PCSK9), which is involved in hepatic function and lipid regulation, increased in cerebrospinal fluid in female and male patients with AUD (Lee et al., 2019). PCSK9 appears to be involved in neuroinflammation [17], and a PCSK9 inhibitor reduced reactive microglia levels by inhibiting the pro-inflammatory NF-κB, suggesting a novel target to treat male and female AUD patients (Apaijai et al., 2019). This body of preclinical work suggests that microglia play an important role in mediating neuroinflammation and influence alcohol-associated behaviors. However, translation work using minocycline has provided inconclusive results in male and female human subjects (Petrakis et al., 2019). Minocycline, although safe, has no effect on alcohol responses in heavy alcohol drinking subjects versus placebo controls (Petrakis et al., 2019). Future work is required to examine the potential use of other microglia acting therapeutic strategies for neuroimmune-related AUD.

### 1.6 Astrocyte evidence

In addition to cytokines and microglial cells, astrocytes have also been reported to be critical regulators of alcohol intake (Carlson et al., 2023; Miguel-Hidalgo, 2018; Adermark and Bowers, 2016). Astrocytes participate in the regulation of innate and adaptive immune responses in the brain (Colombo and Farina, 2016). Astrocytes are dynamic, maintain homeostasis, and like microglia respond to tissue damage that is caused by exogenous agents in the CNS. Neuroimmune response of astrocytes are characterized by their cytoskeletal protein glial fibrillary (GFAP) integrity and analyzed by measuring GFAP immunoreactive levels. Increased GFAP immunoreactive levels are found in multiple astrocyte populations following LPS-induced neuroinflammation (Adermark and Bowers, 2016; Villarreal et al., 2021). Importantly, transcriptional analysis of GFAP + reactive astrocytes can express various cytokines (Orre et al., 2014; Azzolini et al., 2022). However, we recognized that GFAP levels are not broadly expressed in all astrocytes in the brain, are differentially expressed across brain regions, and influenced by age (Kane et al., 2014; Hol and Pekny, 2015; Matias et al., 2019). In humans, alterations of astrocyte markers have been identified in postmortem brain tissue following long-term alcohol use and withdrawal (Kane et al., 1996; Johnson et al., 2015). Alcohol can also activate various immune receptors (e.g., TLRs, cytokine and chemokine receptors) within astrocytes, which also provide overlap in signaling targets and mechanisms to control compromises in neuronal function (Erickson et al., 2019).

There is evidence to suggest that sex and age differences in astrocytes play a role in the regulation of alcohol drinking. Rintala et al. (2001) reported the different expression of GFAP levels, the major intermediate filament protein of astrocytes, in cerebellar folia in young and old male and female rats after 21 months of daily alcohol as the only fluid intake. Indeed, the alcohol-consuming aged female group exhibited a reduction of GFAP in cerebellar folia (II-X) compared with female control rats, whereas their respective aged matched male groups exhibited no differences (Rintala et al., 2001). Wilhelm et al., 2016 also reported activation of GFAP in the HPC and decreased S100β in females versus males (Wilhelm et al., 2016). This report also observed female primary cultured astrocytes exhibited increased expression of *Tnf*, and a reduction in the expression of the neuroprotective cytokine, *Tgfb1*, and reduced excitatory amino acid uptake following alcohol exposure (Wilhelm et al., 2016).

Furthermore, an increase in GFAP immunostaining and morphological alterations of astrocytes were observed in the HPC in both males and females after 10 days of oral alcohol administration by gavage (Kane et al., 2014). Similarly, using a low-fat Lieber-DeCarli liquid diet (5% w/v alcohol) combined with fear-conditioned stress increased GFAP mRNA expression in the HPC and amygdala in male rats (Barkell et al., 2022). Astrocytic glutamate transporter (GLAST) knockout mice exhibited decreased alcohol consumption and preference in a two-bottle free-choice paradigm in both sexes (Karlsson et al., 2012). Furthermore, alcohol treatment increased GFAP levels in female mice compared with male mice, suggesting a greater effect of alcohol-induced astrogliosis in female mice (Alfonso-Loeches et al., 2013). Recently, Brewton et al. (2023) observed sex differences in GFAP expression in models of chronic intermittent ethanol (CIE) vapor exposure and binge-like alcohol drinking (DID). Females exhibited a decrease in GFAP + cells in the bed nucleus of the stria terminalis (BNST) compared with males in the CIE model, whereas no sex differences were observed in the BNST in the DID model (Brewton et al., 2023). To our knowledge, there is no direct evidence linking estrous/hormonal status and astrocyte changes following alcohol exposure.

Astrocytes are an important source of MCP1/CCL2 in the CNS both under normal conditions and during alcohol exposure (Zhang and Luo, 2019; Zhang et al., 2018). Male and female transgenic mice with high levels of MCP1/CCL2 (CCL2-tg) that were subjected to the CIE and two-bottle choice limited-access drinking models exhibited different drinking behaviors between non-dependent and dependent mice (Bray et al., 2017). Non-dependent CCL2-tg females during the two-bottle choice test drank more than males (Bray et al., 2017), whereas no sex differences were observed in the dependent CCL2-tg groups (Bray et al., 2017).

Accumulating evidence of the role of astrocytes in the regulation of synaptic function through direct physical contact (via an extensive network of filament processes) with dendritic spines support the central feature of the astrocyte-neuronal interaction in the maintenance of glutamate homeostasis (i.e., the balance between synaptically released glutamate and extrasynaptic glutamatergic tone) (Allen and Eroglu, 2017). In the synapse, the astrocytic clearance of excess glutamate reduces neuronal excitotoxic liability (Allen and Eroglu, 2017). Studies showed that adolescent intermittent alcohol increases basal glutamate release in the NAc, and in the HPC where it is known to be regulated by astrocytes (Carrara-Nascimento et al., 2011; Risher et al., 2015a; Swartzwelder et al., 2016, 2017). Specifically, adolescent intermittent alcohol differentially induced the immunohistochemical expression of GFAP and altered components (e.g., GluN2A, postsynaptic density 95, etc.) of the glutamatergic systems in the dorsal and ventral HPC in a sex- and subregion-specific manner (Risher et al., 2015b; Crews et al., 2019; Swartzwelder et al., 2019; Healey et al., 2021). Thus, differential hippocampal functional changes that are induced by adolescent intermittent ethanol in male and female rodents could reflect compromised hippocampus-mediated spatial learning and other behavioral phenotypes. Other reports examining the NAc, have shown that stimulation of astrocytes reduces motivation to drink alcohol after abstinence, and shifts reward thresholds using intracranial self-stimulation methods, suggesting a viable mechanism for astrocytes to modulate alcohol drinking in rodents (Bull et al., 2014).

In summary, alcohol exposure disrupts astrocyte signaling and morphology. Females displayed reduced filament protein of astrocytes (GFAP) in the cerebellum and BNST after alcohol exposure as compared to their respective male controls (Rintala et al., 2001; Brewton et al., 2023). Age development may also play a role in sex differences in astrocyte signaling and alcohol since adolescent intermittent alcohol exposure differentially changes GFAP levels and components of the glutamatergic system in the dorsal and ventral HPC (Risher et al., 2015b; Swartzwelder et al., 2019; Healey et al., 2021). Astrocytes represent a potential therapeutic target to alleviate AUD-related neuroimmune responses. However, pharmacological intervention poses a challenge due to overlap of similar immune receptor components (TLRs, cytokines and chemokines) between astrocytes and neurons. Future work regarding sex differences and astrocyte modulation of alcohol requires identification of novel but viable mechanisms to treat AUD.

### 1.7 Synaptic evidence

Neuroimmune signaling refers to communication between the CNS and immune system, which plays a crucial role in regulating various physiological and pathological processes. Synaptic mechanisms are involved in this communication process, with several key neurotransmitters and neuromodulators that act as signaling molecules. For example, cytokines that are released by immune cells modulate synaptic activity, and such neurotransmitters like dopamine, GABA, and glutamate can regulate immune cell function (for review, see (Roberto et al., 2018; Erickson et al., 2019)). Alcohol exposure can disrupt these synaptic mechanisms of neuroimmune signaling, leading to a range of physiological changes in local synaptic transmission in addiction-related neurocircuitry (Roberto et al., 2018, 2021). Specifically, alcohol can affect brain areas that are involved in AUD, including the PFC, amygdala, HPC, NAc, and cerebellum (Roberto et al., 2018; Erickson et al., 2019). Here, we highlight concepts of local synaptic transmission that are affected by humoral (cytokine) and cellular (microglial) components of the neuroimmune response in the latter brain regions in the context of AUD. Importantly, sex differences in this area are significantly understudied, and direct comparisons between males and females including hormone cyclicity in the neuroimmune regulation of neural activity and transmission following alcohol exposure are lacking.

Prior work examined effects of cytokines and microglia on local GABAergic neurotransmission in the CeA, a critical region that is known to mediate stress, anxiety, and alcohol-related behaviors (Roberto et al., 2018; Gilpin et al., 2015; Roberto and Varodayan, 2017; Koob and Volkow, 2016). IL-1β can selectively recruit either neuroprotective phosphoinositide 3-kinase/Akt pathway (PI3K/Akt) or pro-inflammatory (MyD88/p38 MAPK) mechanisms to produce opposing synaptic effects in mPFC (Varodayan et al., 2023). In alcohol naïve conditions, there was a strong PI3K/Akt bias leading to a disinhibition of pyramidal neurons. Alcohol dependence produced opposite IL-1 effects - enhanced local GABAergic inhibition via a switch in IL-1β signaling to the canonical pro-inflammatory MyD88 pathway (Varodayan et al., 2023). Acute alcohol facilitates GABA transmission in a vast majority of CeA neurons in wildtype mice, whereas in IL-1 receptor antagonist (*Il1rn*) knockout mice, this effect was observed only in half of the CeA cells, indicating that IL-1 receptor antagonism contributes to effects of alcohol on CeA inhibitory transmission (Bajo et al., 2015). These findings strongly indicate that effects of IL-1β on CeA GABAergic transmission are mediated by IL-1R1 and potentially through indirect actions of IL-1β via other signaling molecules. Notably, we found similar IL-1β modulation of CeA GABAergic transmission in non-human primates (Patel et al., 2022a).

Bath application of anti-inflammatory IL-10 (50 ng/ml) in alcohol-dependent mice normalized the chronic alcohol-induced upregulation of GABA transmission in the CeA (Patel et al., 2021). These effects were unique to the CeA, in which we did not observe effects of IL-10 on GABAergic transmission in the mPFC in naive or dependent mice despite their similar expression of IL-10 receptors (Patel et al., 2021). Notably, the inhibitory effect of IL-10 in decreasing action potential GABA transmission was observed in CeA neurons from both male and female naïve mice (Patel et al., 2021). Other studies showed that IL-10 modulates GABA transmission in the HPC (Suryanarayanan et al., 2016). Reduced dopamine firing rates in the VTA has been observed in LPS-treated mice versus controls (Blednov et al., 2011). Similarly, immune factors that are recruited during alcohol dependence (e.g., TNF-α, IL-6, BDNF, etc.) have been shown to modulate inhibitory and excitatory synaptic transmission via the modulation of neurotransmitter release and receptors (Roberts et al., 2019; Li et al., 2022; Peregud et al., 2023; Coleman and Crews, 2018).

A recent study reports that IL-18 application decreases vesicular CeA GABA release in male rats exposed to voluntary alcohol drinking, but not in male rats exposed to both stress and alcohol drinking (Borgonetti et al., 2023b). In contrast, in females the magnitude of the IL-18 presynaptic effects on vesicular GABA release is similar across the drinking only and comorbid drinking and stressed rats (Borgonetti et al., 2023b). Notably, female rats with a history of alcohol drinking also displayed IL-18-induced significantly decreases in GABA_A_ postsynaptic functions (an effect absent in male rats with drinking history) and was blunted by the stress exposure (Borgonetti et al., 2023b). Note that this study also lacks assessments of hormone cyclicity.

Microglia also plays a significant role in the modulation of synaptic transmission following alcohol exposure. The alcohol-induced dysfunction of microglia pruning might be a contributor to synapse loss and subsequent memory and cognitive impairments following chronic alcohol intake (Verkhratsky et al., 2021). Our prior work showed that deleting microglia using a CSF1 receptor inhibitor (PLX5622) in alcohol-dependent male mice reduced inhibitory GABA_A_ receptor and excitatory glutamate receptor-mediated synaptic transmission in CeA neurons (Warden et al., 2020). CSF1 viral overexpression during alcohol withdrawal decreased postsynaptic glutamate transmission in mPFC corticotropin-releasing factor-positive neurons, supporting a microglia-mediated mechanism that underlies synaptic adaptations during alcohol withdrawal (Patel et al., 2022b). Notably, chronic alcohol exposure induced abnormal synaptic pruning in the PFC through microglia (Socodato et al., 2020). In the HPC, the substantial loss of excitatory synapses, decreases in the expression of synaptic proteins and glutamate receptor subunits, lower dendrite spine density, and impairments in hippocampal long-term potentiation have been observed following alcohol exposure (Lan et al., 2022). Brain region- and age-dependent differences have been reported for alcohol-induced changes in microglia pruning, whereas sex differences in microglia-mediated effects on synaptic transmission following alcohol exposure have been understudied (Walter and Crews, 2017).

Overall, synaptic mechanisms of neuroimmune signaling and alcohol exposure are complex and involve a wide range of molecular and cellular processes that can contribute to the pathogenesis of AUD.

### 1.8 Behavioral evidence

Animal studies have shown that neuroimmune modulation can significantly affect alcohol drinking behavior. Cytokines, microglia, and astrocytes can alter synaptic functions to play a key role in regulating alcohol intake, and targeting these neuroimmune markers could be a potential therapeutic approach for AUD. For instance, IL-10 plays a role in controlling drinking in male mice (Patel et al., 2021). Whole-brain flow cytometry analysis revealed that IL-10 and IL-10 receptor levels are altered in several cell types, including microglia and astrocytes (Patel et al., 2021). A decrease in IL-10 protein abundance was observed after alcohol dependence in male mice, and (low) IL-10 and (high) IL-1β was positively correlated with baseline alcohol intake (Patel et al., 2021). The overexpression of IL-10 in the CeA decreased anxiety-like behavior and abolished escalation of alcohol intake (Patel et al., 2021). Additionally, bilateral infusions of IL-10 in the basolateral amygdala but not the CeA decreased binge-like alcohol drinking and blood alcohol levels in male mice (Marshall et al., 2017). Another study from our group investigated the role of microglia in alcohol drinking on male mice. The depletion of microglia prevented the dependence-related escalation of alcohol intake (Warden et al., 2020), altered mPFC transcriptome and gene expression pathways associated with GABAergic and glutamatergic signaling, as well as reduced GABAergic and glutamatergic transmission in the CeA (Warden et al., 2020). Although our prior work was performed in male mice, subsequent studies are evaluating effects of these neuroimmune makers in alcohol-dependent female mice. One report examining pleiotrophin (Ptn), a cytokine that inhibits receptor protein tyrosine phosphate (RPTP) β/ζ found that MY10, an RPT RPTP β/ζ inhibitor decreases *Ptn* transcript levels after LPS-induced neuroinflammation and alcohol exposure in female but not male mice (Rodriguez-Zapata et al., 2023).

Gene expression studies found that interleukin-1 receptor type 1 (IL-1R1) is part of a pathway that is associated with a genetic predisposition to high alcohol consumption. A lack of endogenous IL-1 receptor antagonist (IL-1ra) significantly reduced alcohol intake in mice (Blednov et al., 2015). Blednov et al. reported that the deletion of *Il1rn* (which encodes IL-1ra) reduced the severity of acute alcohol withdrawal and increased sensitivity to sedative/hypnotic effects of alcohol and flurazepam in both males and females (Blednov et al., 2015). In contrast, *Il1r1* deletion increased the severity of acute alcohol withdrawal and reduced sensitivity to sedative effects of alcohol and flurazepam (Blednov et al., 2015). The administration of Kineret (IL-1ra) attenuated the increase in alcohol- and flurazepam-induced sedation in *Il1rn* knockout mice, and pretreatment with Kineret restored the severity of acute alcohol withdrawal (Blednov et al., 2015). Only female *Il1r1* KO mice displayed quicker recovery to baseline from alcohol-induced motor incoordination (Blednov et al., 2015). Mice that lacked *Il1rn* but not *Il1r1* exhibited an increase in alcohol clearance and a decrease in alcohol-induced conditioned taste aversion (Blednov et al., 2015). In both null mutants, alcohol-induced sedation and withdrawal severity were affected in opposite directions, suggesting the involvement of IL-1R1 signaling in sedation and withdrawal (Blednov et al., 2015).

Toll-like receptor signaling has also been shown to be influenced by alcohol drinking. Several TLRs have been implicated in rodent models of alcohol drinking (Blednov et al., 2011, 2017b). Toll-like receptors can produce the release of several cytokines (Blasius and Beutler, 2010). For example, TLR3 are increased after chronic alcohol exposure and activation of TLR3 and TLR7 increases alcohol consumption in male mice (Warden et al., 2019a; Grantham et al., 2020). In females, TLR3 activation decreases alcohol intake at a point that corresponds to high levels of innate immune activity, an effect dependent on the presence of MyD88 (Warden et al., 2019b). Interestingly, TLR3 agonist administration increased alcohol intake, an effect that was greater in females than males at lower doses (Lovelock et al., 2022; Randall et al., 2019). Repeated injections of TLR7 agonist increases operant alcohol self-administration with no sex differences in responses for alcohol (Lovelock et al., 2022). TLR4 deficiency in mice prevented the increases in mRNA levels of TNF-α, IL-1β, and IL-6 in the cortex of female mice, suggesting a role for TLR4 in cytokine release following alcohol exposure (Alfonso-Loeches et al., 2010). A TLR 4 inhibitor has shown to decrease alcohol drinking and IDA-1 expression in the CeA of alcohol dependent male mice (Bajo et al., 2016). Viral-mediated knockdown of TLR4 in the CeA and ventral tegmental area but not the ventral pallidum decreased binge-like alcohol drinking in mice (Liu et al., 2011; June et al., 2015). However, deletion of TLR4 had minimal effect on alcohol drinking in rodents (Harris et al., 2017).

Anti-inflammatory drugs that interact with enzymes that act on phosphodiesterase-4 (PDE4) have also shown promising therapeutic effects in reducing alcohol drinking and negative emotional states that are associated with alcohol abstinence. Ibudilast, an anti-inflammatory drug with PDE4-inhibiting properties, decreased alcohol intake across a range of doses and drinking paradigms in male alcohol-dependent mice (Bell et al., 2015). Other studies described similar reductions of alcohol drinking in mice and rats using rolipram (Hu et al., 2011; Wen et al., 2012). Rolipram also reduced alcohol intake and blood alcohol levels in both male and female High DID-1 (HDID-1), HDID-2, and Heterogenous Stock/Northport mice (Ozburn et al., 2020). In humans, ibudilast reduced alcohol cue-included functional connectivity in the orbitofrontal and anterior cingulate cortices, suggesting a lower likelihood of relapse in ibudilast-treated individuals (Burnette et al., 2021). Importantly, ibudilast has shown promising efficacy in reducing drug intake in females for other drugs of abuse (Poland et al., 2016). Apremilast, originally approved by the Food and Drug Administration for psoriasis with high repurposing potential for AUD, displayed effectiveness in decreasing binge-like alcohol drinking in mice that were selectively bred for high alcohol intoxication (Grigsby et al., 2023). Apremilast at 20 and 40 mg/kg decreased alcohol intake in stressed alcohol-dependent mice (Grigsby et al., 2023). This study also found that human participants with AUD that received apremilast (90 mg/day) drank significantly less alcohol than placebo controls via fewer drinks per day and probably less heavy drinking during an 11-day regimen of *ad libitum* access (Grigsby et al., 2023). The changes that were observed in this report considered both males and females, showing the promising efficacy of apremilast in reducing drinking behavior in both sexes. The effects of apremilast appear to be driven by the excitation of dopamine D_1_ but not D_2_ medium spiny neurons in the NAc (Grigsby et al., 2023). Similarly, a recent study suggests the effects of apremilast in modulating alcohol drinking occur via PKA-dependent GABAergic signaling. The reduction of alcohol intake by apremilast is prevented in β3-S408/409A male and female mice, a mouse genetic line that prevents PKA-dependent phosphorylation of β3 subunits (Blednov et al., 2022). PDE4 subtypes B and D also modulate alcohol drinking in male and female mice. PDE4-D inhibitors transiently decreases alcohol drinking, while PDE4-B has no effect on drinking in either sex (Blednov et al., 2023). Another report examined 4 different immune related compounds (tacrolimus, sirolimus, palmitoylethanolamide, [PEA] and secukinumab) in a HDID-1 drinking mice model in both males and females (Grigsby et al., 2020). Out of the 4 immune-related drugs, tacrolimus reduced alcohol intake and BALs in both male and female HDID-1 mice across various doses (Grigsby et al., 2020). Sirolimus, PEA, and secukinumab showed no significant changes in alcohol drinking or unique sex differences in responding for alcohol in male and female HDID-1 mice (Grigsby et al., 2020). An important consideration is the lack of estrous/hormonal assessments that might explain differences in the behavioral output of alcohol related behaviors observed in these studies. Future work is needed to fully characterize estrous/hormonal status with neuroinflammation and alcohol-associated behaviors.

## 2 Conclusion

A summary of effects on neuroimmune-related agents and alcohol drinking with underlying sex differences is indicated in Table 1. In summary, the findings in this review support the notion that alcohol affects various aspects of neuroimmune signaling and synaptic function distinctly in males and females. Chronic alcohol exposure can lead to neuroinflammation, which is partially characterized by the activation of microglia and astrocytes and release of cytokines with unique sex differences. This disruption in cytokines, microglia, and astrocytes can directly affect synaptic function and plasticity by altering the release and uptake of neurotransmitters, modulating ion channels, and disrupting the structure and function of synapses. Animal studies demonstrate a link between neuroimmune responses and alcohol intake. Genomic and transcriptomic findings suggest the rewiring of neuroimmune genetic circuits following chronic alcohol intake with distinct sex differences. Electrophysiological data suggest that synaptic transmission and neuronal circuitry are affected by neuroimmune targets in the brain in males, which provides a mechanistic framework for future studies in females in alcohol dependence. It is also important to consider the complication of identifying unique neuroimmune circuitry to influence alcohol-associated behaviors given the challenges in unraveling cross talk between CNS and PNS sources (e.g., peripheral organs and circulating blood). Peripheral immune to brain communication is affected by transport and trafficking of immune cells through the blood brain barrier, afferent and efferent communication between CNS and PNS, providing a challenge in distinctly identifying specific signaling or inflammatory mediators from both or one another with reciprocal interactions to influence circuits and behaviors of alcohol use (Dantzer, 2018). Peripheral immune cell also activate nerve afferents and are activated by sensory afferents and autonomic efferents (Shouman and Benarroch, 2021). Overall, these findings highlight the importance of understanding the effects of alcohol on neuroimmune signaling, synaptic function, and sex differences to identify potential therapeutic targets for AUD.

**Table 1:** Summary of neuroinflammatory agents on brain and behavioral effects of alcohol use and underlying sex differences.

Drug Treatment	Mechanism of Action	Species/Subjects	Age	Dose & Route of Administration	Brain Region	Synaptic/Cellular/Molecular Effects	Behavioral Effects	Reference
Minocycline	Microglial suppressor, binds to bacterial 30S ribosomal subunit and interferes protein synthesis	Male & Female C57Bl/6J Mice	Adult	50 mg/kg; IP Injection	–	–	Decrease alcohol intake in both male and female mice	Agrawal et al., 2011; Agrawal et al., 2014
Minocycline	Microglial suppressor, binds to bacterial 30S ribosomal subunit and interferes protein synthesis	Male & Female Human Heavy Drinkers	Adult	100 mg/day or 200 mg/day; Oral Administration		–	No effect in alcohol drinking in either sex	Petrakis et al., 2019
Minocycline	Microglial suppressor, binds to bacterial 30S ribosomal subunit and interferes protein synthesis	Male & Female C57Bl/6J Mice	Adolescent	50 mg/kg; IP Injection	–	–	No effect in alcohol drinking in either sex	Agrawal et al., 2014
Tigecycline	Binds to a helical region (H34) on the 30S subunit of bacterial ribosomes	Male & Female DBA/2J Mice	Adult	0,20,40, or 80 mg/kg; IP	–	–	Decrease in both sexes for alcohol withdrawal induced convulsion	Martinez et al., 2016
Tigecycline	Binds to a helical region (H34) on the 30S subunit of bacterial ribosomes	Male & Female DBA/2J Mice	Adult	0, 20, 40, 60, 80, 100, 120 mg/kg; IP	–	–	Decrease in sexes for alcohol withdrawal induced convulsion	Syapin et al., 2016
Tigecycline	Binds to a helical region (H34) on the 30S subunit of bacterial ribosomes	Male & Female C57Bl/6J Mice	Adult	80 mg/kg; IP	–	–	Antinociceptive effects in males; pro‐nociceptive in females. Reduced mechanical and cold sensitivities in males, but increased females	Bergeson et al., 2016
IL-10	Decrease cytokine T-cell proliferation, inhibits tyrosine phosphorylation of CD28 and phosphatidylinositol 3-kinase binding	Male C57Bl/6J Mice	Adult	0, 100 ng intracranial infusion	BLA & CeA	–	IL-10 in the BLA but not the CeA decreased binge-like alcohol drinking and blood alcohol levels in male mice	Marshall et al., 2017
Il-1Ra antagonist	Blocks Il-1Ra and inhibits Il-1 signaling	Male C57BL/6J mice	Adult	0.9 μg IL-1Ra	BLA & CeA	–	IL-1Ra antagonist infusions in the BLA but not CeA decreased alcohol consumption	Marshall et al., 2016
Kineret	IL-1ra receptor antagonist blocks Il-1α and Il-1β	Male *Il1rn* Ko & WT mice from C57BL/6J Bakcground	Adult	100 mg/kg; IP	–	–	Kineret (IL-1ra) attenuated the increase in alcohol- and flurazepam-induced sedation in *Il1rn* knockout mice, and restored the severity of acute alcohol withdrawal	Blednov et al., 2015
polyinosinic-polycytidylic acid (poly (I:C))	TLR3 agonist, activates TLR3	Male C57Bl/6J Mice	Adult	0, 2, 5, or 10 mg/kg, i.p.)	–	–	Increased alcohol intake	Warden et al., 2019a
polyinosinic-polycytidylic acid (poly (I:C))	TLR3 agonist, activates TLR3	Female C57Bl/6J Mice	Adult	5 mg/kg; IP	–	–	TLR3 activation decreases alcohol intake at a point that corresponds to high level of innate immune activity.	Warden et al., 2019b
polyinosinic-polycytidylic acid (poly (I:C))	TLR3 agonist, activates TLR3	Male & Female Long Evan Rats	Adult	0, 0.3, 1.0 mg/kg; IP	–	–	TLR3 agonist administration increased alcohol intake, an effect that was greater in females than males at lower doses	Lovelock et al., 2022
polyinosinic-polycytidylic acid (poly (I:C))	TLR3 agonist, activates TLR3	Male Long Evan Rats	Adult	0, 3 mg/kg, IP	Nac, IC	TLR3 activation increased mRNA levels of neuroimmune genes (TLR3, COX2), glutamatergic genes (mGluR 2, mGluR 3, GLT1), and the trophic factor BDNF in Acb and IC.	TLR3 activation also increased ethanol self-administration 18 days post-injection,	Randall et al., 2019
T5342126	TLR4	Male C57BL/6J mice	Adult		CeA	T5342126 reduced Iba-1 density in the CeA of both alcohol-dependent and non-dependent mice injected with T5342126.	T5342126 decreased alcohol drinking in both dependent and non-dependent mice	Bajo et al., 2016
R848	TLR7 agonist	Male C57BL/6J	Adult	50 μg; IP	PFC, NAc, HPC, Amygdala, VTA,	Acute TLR7 activation produces brain region specific changes in expression of immune pathway genes, whereas chronic TLR7 activation causes downregulation of TLRs and blunted cytokine induction	Acute activation of TLR7 reduced alcohol intake, preference, but chronic pre-treatment with R848 increases in alcohol consumption	Grantham et al., 2020
Ibudilast	phosphodiesterase-4 inhibitor	Male P & HAD1 rats	Adult	0, 3, 6 or 9 mg/kg; SC	–	–	Decreased alcohol intake across a range of doses and drinking paradigms in male alcohol-dependent mice	Bell et al., 2015
Ibudilast	phosphodiesterase-4 inhibitor	Male & Female Human subjects with AUD	Adult	Placebo or 50 mg/BID; Oral	OFC, ACC	reduced alcohol cue-included functional connectivity in the orbitofrontal and anterior cingulate corticessuggesting a lower likelihood of relapse in Ibudilast-treated individuals	–	Burnette et al., 2021
Rolipram	phosphodiesterase-4 inhibitor	Male C57BL/6J	Adult	0.1, 0.25, and 0.5 mg/kg; IP	–	–	Reduced alcohol intake	Hu et al., 2011
Rolipram	phosphodiesterase-4 inhibitor	Male Fawn-Hooded (FH/Wjd) rats	Adult	0, 0.05, 0.1, 0.2 mg/kg; SC	–	–	Rolipram dose-dependently reduced alcohol self-administration	Wen et al., 2012
Rolipram	phosphodiesterase-4 inhibitor	Male & Female HDID-1, HDID-2, & HS/NPT mice.	Adult	0 or 5 mg/kg; PO	–	–	Decreased DID intake and BALs in male and female HDID-1, HDID-2, and HS/NPT mice.	Ozburn et al., 2020
Apremilast	phosphodiesterase-4 inhibitor	Male & Female HDID-1 & HDIDD 2 mice Male C57BL/6J mice	Adult	0, 20, 40 mg/kg; IP	NAc	Apremilast reduced binge-like drinking behavior through increasing excitability of NAc D1, but not NAc D2, MSNs	Decreased alcohol drinking in mice that were selectively bred for high alcohol intoxication and in stressed dependent mice.	Grigsby et al., 2023
Apremilast	phosphodiesterase-4 inhibitor	Male & Female Human Subject with AUD	Adult	Placebo or 90 mg/day; Oral	–	–	Reduced alcohol drinking in both sexes.	Grigsby et al., 2023
Apremilast	phosphodiesterase-4 inhibitor	Male & Female β3-S408/409A heterozygous mice C57BL/6J background	Adult	(20 mg/kg, p.o.)	–	–	The reduction of alcohol intake by apremilast were prevented in β3-S408/409A male and female mice	Blednov et al., 2022
D159687	phosphodiesterase-4 inhibitor selective for subtype D	Male & Female C57Bl/6J Mice	Adult	3 mg/kg; IP	–	–	D159687 transiently decreased alcohol drinking in both sexes	Blednov et al., 2023
A33	phosphodiesterase-4 inhibitor selective for subtype B	Male & Female C57Bl/6J Mice	Adult	1 mg/kg; IP	–	–	No effect on alcohol drinking in either sex.	Blednov et al., 2023
MY10	RPT RPTP β/ζ inhibitor	Male & Female WT *Ptn*+/+ & *Ptn*-Tg C57Bl/6J Mice	Adult	O or 60 mg/kg; oral	–	RPT RPTP β/ζ inhibitor decreased *Ptn* transcript level after LPS induced neuroinflammation and alcohol exposure in females but not male mice	–	Rodriguez-Zapata et al., 2023
Tacrolimus	Macrolide calcineurin inhibitor	Male & Female HDID-1 mice	Adult	0, 0.5, 1, 2 mg/kg; IP	–	–	Tacrolimus reduced alcohol intake and BALs in both sexes.	Grigsby et al., 2020
Sirolimus	mTOR inhibitor, inhibits T-lymphocyte activation	Male & Female HDID-1 mice	Adult	0, 5, 10, or 20 mg/kg; IP	–	–	No effect on alcohol intake in either sex	Grigsby et al., 2020
Palmitoylethanolamide	Activates of proliferator activated receptor alpha (PPARα)	Male & Female HDID-1 mice	Adult	0, 75, 150, 225 mg/kg; SC	–	–	No effect on alcohol intake in either sex	Grigsby et al., 2020
Secukinumab	Binds to the IL-17A cytokine and inhibits its interaction with the IL-17 r.	Male & Female HDID-1 mice	Adult	0, 5, 30, 60 mg/kg	–	–	No effect on alcohol intake in either sex	Grigsby et al., 2020

## 3 Human and animal rights and informed consent

This article does not contain any studies with human or animal subjects performed by any of the authors.

## Funding source

Support was provided by 10.13039/100000027National Institute on Alcohol Abuse and Alcoholism grants AA027700, AA013498, P60 AA006420, AA017447, AA021491, AA029841, and T32 AA007456, the Schimmel Family Endowed Chair, and the Pearson Center for Alcoholism Addiction Research. The authors thank Michael Arends for editing the manuscript. The authors declare no competing financial interests.

## CRediT authorship contribution statement

**Bryan Cruz:** Conceptualization, Writing – original draft, Writing – review & editing, Supervision. **Vittoria Borgonetti:** Conceptualization, Writing – original draft, Writing – review & editing. **Michal Bajo:** Conceptualization, Writing – original draft, Writing – review & editing. **Marisa Roberto:** Conceptualization, Writing – original draft, Writing – review & editing, Supervision, Funding acquisition.

## Declaration of competing interest

All authors have no conflict of interest.

## Data Availability

No data was used for the research described in the article.
